# Small RNAs, big impact: microRNAs in malaria pathogenesis, therapeutic opportunities, and challenges

**DOI:** 10.3389/fcimb.2026.1883875

**Published:** 2026-07-31

**Authors:** Nahla Galal Metwally, Maria del Pilar Martínez Tauler, Hanifeh Torabi, Iris Bruchhaus

**Affiliations:** 1 Research Group Host Parasite Interaction, University of Hamburg, Hamburg, Germany; 2 Biology Department, University of Hamburg, Hamburg, Germany

**Keywords:** brain endothelial cells, lung endothelial cells, malaria, miRNA and mRNA, *Plasmodium falciparum*

## Abstract

Malaria is one of the world’s most critical parasitic diseases; it is caused by *Plasmodium* species and spreads through *Anopheles* mosquitoes. The life cycle of *Plasmodium* species is complex and involves several stages in humans and mosquitoes, each involving distinct molecular interactions. Recent molecular biology research has suggested that host microRNAs (miRNAs) may serve as important regulators of host–parasite interactions during malaria infection. MicroRNAs, approximately 22 nucleotides long, are non-coding RNAs that control gene expression by binding to target mRNAs and either repressing translation or causing mRNA degradation. During infection, host miRNAs are actively regulated and can directly target parasite transcripts or influence host cellular pathways essential for parasite survival and disease development. The role of human miRNAs has not yet been fully explored in malaria. This report aims to integrate evidence from the literature, molecular mechanisms, and translational insights to summarize the current knowledge in miRNA research and to guide the diagnosis and treatment of malaria using miRNAs. Here, we focus on the mechanistic and cell-type-resolved dimension of host miRNA action in malaria: the extracellular vesicle-mediated transfer of AGO2–miRNA complexes between infected erythrocytes and recipient endothelial cells, the bidirectional host–parasite miRNA traffic that allows human miRNAs to repress parasite transcripts, and the tissue-specific miRNA programs that shape organ-specific pathology. We also delineate the principal knowledge gaps related to causality versus correlation, cell-of-origin attribution, and translational barriers to miRNA therapeutics that must be resolved before miRNAs can be deployed diagnostically or therapeutically in malaria.

## Highlights

- Host miRNAs are dynamically remodeled across the liver and blood stages and during severe malaria, where they converge on immune, endothelial, and red-cell stress pathways.
- Extracellular vesicles from infected erythrocytes deliver functional miRNA cargo to recipient cells, linking parasitic infection to vascular activation and barrier dysfunction.
- A subset of host miRNAs can enter *P. falciparum* and repress parasite transcripts, providing a potential route for direct antiparasitic strategies.
- Circulating/EV miRNA signatures show promise for severity stratification but require rigorous control of hemolysis, platelet contamination, and normalization.
- Therapeutic translation hinges on delivery, off-target/immune activation risks, and identifying causal miRNA–target pairs in relevant cell types.

## 1 Introduction

### 1.1 Pathogenesis of *Plasmodium falciparum* infection

Malaria is caused by protozoan parasites of the genus *Plasmodium*, which are transmitted to humans through the bite of an infected female *Anopheles* mosquito. Inoculated sporozoites first travel to the liver and replicate silently within hepatocytes (the pre-erythrocytic, or liver, stage) before being released into the bloodstream as merozoites. Each merozoite invades a red blood cell and undergoes repeated cycles of asexual replication (the erythrocytic, or blood, stage); it is this blood stage that produces the fever, anemia, and other clinical manifestations of malaria, while a subset of parasites differentiates into sexual gametocytes that sustain onward transmission. Against this shared life-cycle background, *Plasmodium falciparum* is distinguished by its capacity to remodel the infected erythrocyte and drive the severe, often fatal, complications described below.

**Figure 1:**
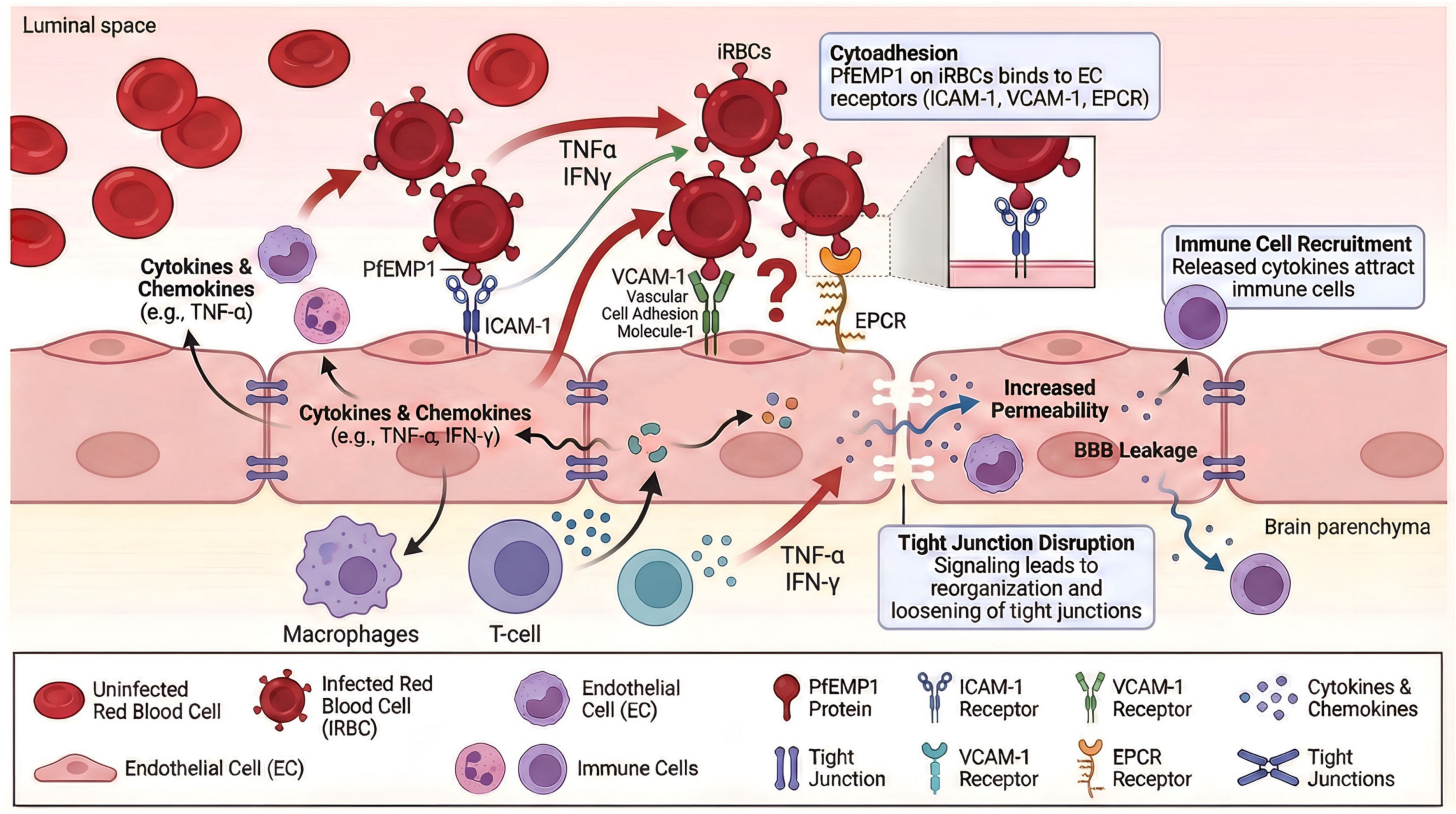
Pathogenic events during *Plasmodium falciparum* infection. Infected red blood cells (iRBCs) interact with brain endothelial cells (ECs). The PfEMP1 protein on iRBCs binds to various receptors on these cells, such as ICAM-1, VCAM-1, and EPCR. Various events, such as cytokine release and immune cell recruitment, activate several signaling pathways in ECs. This activation results in the reorganization of tight junctions and increased permeability of the blood–brain barrier (BBB).

Among the five parasite species that cause malaria, infection with *P. falciparum* is the most lethal and causes the great majority of malaria deaths. Globally, an estimated 282 million malaria cases and 610,000 deaths occurred in 2024, with approximately 95% of deaths in the WHO African Region (WHO, 2025). These deaths result from severe complications of malaria. These include cerebral malaria (CM), lung injury, renal failure, acidosis, and severe anemia (Cunnington et al., 2013). The pathogenesis of *P. falciparum* infection encompasses four principal mechanisms (**Figure 1**) (Taylor et al., 2004; Craig et al., 2012; Cunnington et al., 2013; Milner et al., 2013; Gazzinelli et al., 2014; Phillips et al., 2017; Moxon et al., 2020):

Sequestration: *Plasmodium falciparum* modifies infected red blood cells (iRBCs), causing them to sequester in the microvasculature of vital organs. This interaction leads to microvascular obstruction, impairing perfusion and causing hypoxia, which can result in organ failure and death. A key parasite ligand, *Pf*EMP1, interacts with human endothelial cell receptors (ECRs), mediating cytoadhesion and sequestration. However, sequestration alone does not account for all features of severe malaria, such as rapid recovery after antimalarial treatment, the rarity of tissue infarction despite vessel occlusion, and the fact that severe cases caused by *Plasmodium vivax* (*P. vivax*) cause less sequestration.

Inflammatory response (evidence from non-*P. falciparum* malaria): A highly active or dysregulated inflammatory response is a key factor in severe malaria. Studies have shown elevated levels of inflammatory mediators in infected patients compared with those in uncomplicated malaria patients, indicating a role for immunopathology in experimental cerebral malaria in mice. *P. vivax* triggers a stronger proinflammatory response per parasite than does *P. falciparum*, possibly explaining severe disease. Cytokines and mediators from leukocytes may directly damage organs, interact with molecules such as free heme, or cause indirect harm via the endothelium, mitochondria, or metabolic pathways.

Disseminated intravascular coagulation: Widespread activation of the coagulation system leads to severe complications such as cerebral thrombosis, hemorrhage, intracerebral hypoxia, and reduced blood flow. Moxon et al. suggested that intravascular coagulation in CMs may be caused by reduced expression of the iRBC-binding receptor EPCR and the anticoagulant/thrombin receptor thrombomodulin (TM). The coagulation cascade is triggered by iRBCs, which activate both the extrinsic and intrinsic pathways by inducing ECs to express tissue factor (TF). TF initiates coagulation by converting fibrinogen into fibrin, thereby promoting thrombosis.

Dysregulation of vascular endothelial cells: Activation of the vascular endothelium is increasingly recognized as a key contributor to severe malaria. It can be activated by inflammatory cytokines and materials from iRBCs, such as hemoglobin, glycosylphosphatidylinositol, and histones. Evidence also shows activation of clotting in the microvasculature and impairment of the barrier, enabling cytokines or toxins to interact directly with tissues.

#### 1.1.1 Scope and novelty of this review

Several reviews have already surveyed miRNAs in malaria, most of which focus on circulating miRNAs as candidate diagnostic or prognostic biomarkers (Rubio et al., 2016; Rangel et al., 2020; Kataria et al., 2022). Rather than re-cataloguing these biomarker lists, the present review is organized mechanistically and by cellular compartment and is deliberately weighted toward developments that post-date those earlier syntheses. We focused on three main areas. First, we foreground extracellular vesicle (EV)-mediated delivery of functional Argonaute 2 (AGO2)–miRNA (RISC) complexes from infected erythrocytes to recipient endothelial cells, and the resulting silencing of barrier and inflammatory genes, a route only partially addressed by prior reviews. Second, we treat host–parasite miRNA traffic as bidirectional, covering both the import of human miRNA–RISC complexes into *P. falciparum* and the parasite-side adaptations that may blunt this regulation. Third, we emphasize tissue-resolution contrasts in brain– and lung–endothelial miRNA/mRNA responses to infected erythrocytes, arguing that organ-specific miRNA programs, rather than a single circulating signature, underlie the distinct syndromes of severe malaria. Throughout, we separate what has been demonstrated causally from what remains correlative, and we close by mapping the specific experimental and translational gaps that must be addressed before miRNAs can be used clinically.

### 1.2 Biogenesis and release of miRNAs

**Figure 2:**
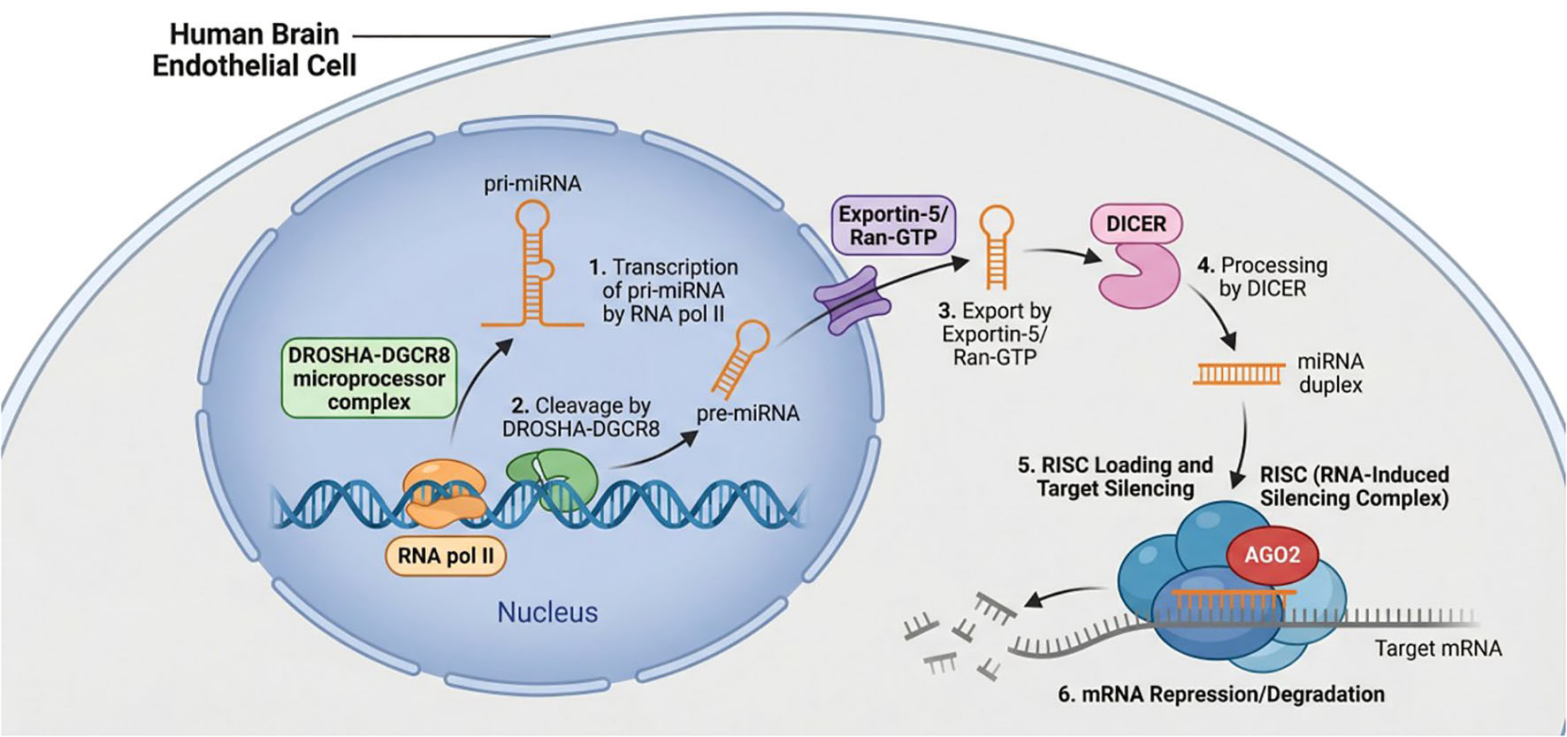
MicroRNA biogenesis. The process begins with the creation of the pri-miRNA transcript. The microprocessor complex, composed of Drosha and DGCR8, cleaves pri-miRNA to generate pre-miRNA. This pre-miRNA is exported to the cytoplasm in an Exportin-5/Ran-GTP-dependent manner and processed into the mature miRNA duplex. The 5p or 3p strand of this duplex is then loaded into Argonaute (AGO) proteins to form the miRNA-induced silencing complex (miRISC). In non-canonical pathways, small hairpin RNAs (shRNAs) are initially cleaved by the microprocessor complex and exported via Exportin-5/Ran-GTP. These pre-miRNAs are further processed by AGO2-dependent, Dicer-independent cleavage. Functional miRISC binds target mRNAs to inhibit translation.

MicroRNAs (miRNAs) are small non-coding RNAs consisting of 19 to 24 nucleotides (Ha and Kim, 2014). miRNA biogenesis occurs through canonical and non-canonical pathways that together ensure versatile posttranscriptional gene regulation. In the canonical pathway, miRNA genes are transcribed by RNA polymerase II into capped and polyadenylated primary transcripts (pri-miRNAs), which are cleaved in the nucleus by the Drosha-DGCR8 microprocessor complex into precursor miRNAs (pre-miRNAs). These pre-miRNAs are exported to the cytoplasm by Exportin-5/Ran-GTP and further processed by Dicer to produce ~22-nucleotide miRNA duplexes, one strand of which is loaded into Argonaute (AGO) to form the RNA-induced silencing complex (RISC), which represses target mRNAs via translational inhibition or degradation (**Figure 2**) (Ha and Kim, 2014). In contrast, non-canonical pathways bypass one or more of these steps, allowing miRNA formation under specialized or stressed conditions. Mirtrons are splicing-derived short introns that fold into pre-miRNA hairpins independently of Drosha, while AGO2-dependent maturation processes certain pre-miRNAs, such as miR-451, without Dicer (Cheloufi et al., 2010; Meijer et al., 2014). Other non-canonical routes use snoRNAs, tRNAs, or endogenous short hairpin RNAs (shRNAs) as substrates, producing functional miRNA-like molecules that participate in adaptive and compensatory gene regulation during stress, hypoxia, or infection (**Figure 2**) (Gu et al., 2009; Cheloufi et al., 2010; Yoda et al., 2010; Yang and Lai, 2011; Havens et al., 2012; Meijer et al., 2014).

Once loaded into the RISC complex, the miRNA guides the complex to its target mRNA by base pairing with complementary sequences in the 3′ untranslated region (3′ UTR) (O'Brien et al., 2018). Perfect or near-perfect complementarity (mainly in plants): This process leads to mRNA cleavage and degradation via AGO2-mediated endonucleolytic activity. Imperfect complementarity (common in animals): This process leads to translational repression and mRNA destabilization through the inhibition of translation initiation (blocking cap recognition or ribosome assembly) and the deadenylation of the mRNA’s poly(A) tail, reducing stability and decapping and subsequent 5′→3′ exonucleolytic degradation (Valinezhad Orang et al., 2014). Several key factors affect miRNA function: First, the seed region (nucleotides 2–8) determines target specificity; mismatches in this region can abolish binding. Second, with respect to target site accessibility, the secondary structure of mRNAs affects miRNA binding. Third, competing endogenous RNAs (ceRNAs): Other RNAs (e.g., lncRNAs and circular RNAs) can “sponge” miRNAs, reducing their regulatory effects. Finally, subcellular localization: Several miRNAs localize to specific cellular compartments, thereby influencing target selection (Zheng et al., 2025).

Various cell types release miRNAs through multiple mechanisms, including the following:

- Passive release (cell damage or apoptosis): Damaged or dying cells release naked miRNAs or miRNA-protein complexes into the extracellular space [these miRNAs are often associated with AGO2 or nucleophosmin 1 (NPM1), which protect them from degradation by extracellular RNases].
- Active secretion (regulated pathways): Cells can actively export miRNAs via vesicle-dependent and vesicle-independent pathways:
- Exosomes: Lipid vesicles (30–150 nm) are formed in multivesicular bodies, and miRNAs are selectively packaged and released through exocytosis.
- Microvesicles (MVs): Vesicles (100–1,000 nm) shed from the plasma membrane contain cytoplasmic components, including miRNAs, reflecting the state of the donor cell.
- Apolipoprotein-bound miRNAs: HDL/LDL particles and miRNAs associate with lipoproteins for long-range transport in the bloodstream.
- Ribonucleoprotein complexes: AGO2 or NPM1 bind to the non-vesicular form, stabilizing the protein against RNases (Iftikhar and Carney, 2016; O'Brien et al., 2018).

Once released, ex-miRNAs can be taken up by recipient cells via vesicle endocytosis, exosome-mediated membrane fusion, or receptor-mediated binding to surface proteins (e.g., TLR7 and TLR8). Inside the recipient cell, ex-miRNAs, similar to endogenous miRNAs, enter the RISC and regulate gene expression posttranscriptionally (Ortiz-Quintero, 2020).

## 2 Role of human miRNAs in malaria pathogenesis

### 2.1 Human miRNA profiles during liver stages

During the silent liver stage, *Plasmodium* sporozoites invade hepatocytes and develop into exoerythrocytic forms (EEFs). This process involves host miRNA reprogramming, which influences both parasite development and host immunity. In a mouse model of *P. berghei* liver infection, Hammerschmidt-Kamper observed a transcriptional decrease in key miRNA/RNAi components, including Exportin-5, AGO2, Dicer, TRBP, and Drosha, especially approximately 40 h after infection with attenuated parasites. They also identified 31 host hepatic miRNAs whose expression varied with the parasite strain and attenuation method (wild-type, genetically attenuated, or irradiated sporozoites). Notably, miR-21 and miR-155 were confirmed to be overexpressed in infected liver tissue. Functional *in vivo* knockdown experiments indicated that both miRNAs contribute to the protective immunity induced by genetically attenuated parasites. Mechanistically, these conclusions rest on complementary gain- and loss-of-function experiments in the rodent model rather than on expression profiling alone. *In vivo* antagomir-mediated knockdown of miR-21 or miR-155 in the livers of mice immunized with genetically attenuated sporozoites reduced the protective effect, while AAV8-driven hepatic overexpression of miR-155 enhanced the protection conferred by attenuated parasites (Hentzschel et al., 2014), placing miR-155 upstream of the antiparasitic CD8^+^ T-cell response that mediates liver-stage immunity. The key readouts in these studies were liver parasite burden after sporozoite challenge and sterile protection rates, providing functional, rather than purely correlational, support for a role for these miRNAs in pre-erythrocytic immunity. Supporting these findings, a review of host–*Plasmodium* RNAi interactions revealed that dysregulated host miRNAs can regulate cellular pathways critical for *Plasmodium* replication and influence host processes such as metabolism and immune signaling during hepatic infection (Hammerschmidt-Kamper, 2012; Shrivastava and Rajasubramaniam, 2018).

### 2.2 Human miRNA profiles during erythrocytic stages

Although human red blood cells (RBCs) are enucleated and devoid of transcriptional activity, they harbor a diverse population of miRNAs that play regulatory roles in erythroid differentiation (Zhan and Song, 2008), oxidative stress control, and intercellular communication via extracellular vesicles (EVs). Despite lacking nuclei, RBCs retain functional AGO2-bound miRNA complexes, enabling posttranscriptional gene regulation even in their terminal form (Perla et al., 2025). Comprehensive RNA-seq and proteomic studies have identified a select set of miRNAs that dominate the mature RBC transcriptome and are critical in both physiological and pathological contexts (Azzouzi et al., 2015; Ryan and Atreya, 2011). For example, miR-451a regulates oxidative stress by suppressing 14-3-3ζ, which is essential for terminal erythroid maturation (Azzouzi et al., 2015). miR-144-3p and miR-144-5p are cotranscribed with miR-451a; they regulate erythroid gene expression and contribute to RBC stability and are often described as “erythroid signature miRNAs” (Noh et al., 2009; de Vasconcellos et al., 2017). They are coregulated by GATA-1 and KLF1, the master transcription factors of erythropoiesis (Rasmussen et al., 2010; Zhu et al., 2013). miR-16-5p is involved in apoptosis and stress signaling and is abundant in both RBCs and platelets. Additionally, miR-486-5p is erythroid-specific and regulates PTEN and FOXO1, influencing erythroid survival and maturation (Juzenas et al., 2020). RBC-derived EVs are rich in miR-451a, miR-486-5p, and miR-92a, indicating their role in cell–cell signaling and immune modulation (Bonauer et al., 2009; Joshi et al., 2025). **Table 1** lists important human miRNAs in RBCs. Of these, miR-451a and let-7i-5p are the most relevant to malaria: both can enter the parasite to inhibit translation, and EV-borne forms additionally temper endothelial inflammation, whereas miR-16-5p is of interest mainly as a circulating biomarker. The remaining entries (miR-144, miR-486-5p, miR-92a) act chiefly in erythroid maturation and EV-mediated signaling.

**Table 1:** Important human miRNAs in red blood cells.

miRNA	Principal target(s)	Function in RBCs/relevance to malaria	Key reference
miR-451a	14-3-3ζ; modulates autophagy	Master regulator of erythroid maturation and oxidative-stress control; mimic reduces parasitemia and oxidative stress in *P. falciparum*-iRBCs; enriched in sickle-cell RBCs	(Yu et al., 2010; LaMonte et al., 2012; Joshi et al., 2024)
miR-144-3p/miR-144-5p	Erythroid gene network (co-regulated with miR-451a)	Co-transcribed with miR-451a from the miR-144/451 locus; contribute to RBC stability; “erythroid signature” miRNAs	(Rasmussen et al., 2010)
miR-16-5p	Apoptosis/stress-signaling genes	Abundant in RBCs and platelets; consistently downregulated in plasma of malaria patients (candidate biomarker)	(Chamnanchanunt et al., 2015)
miR-486-5p	PTEN; FOXO1	Erythroid-specific; promotes erythroid survival and maturation; enriched in RBC-derived EVs	(Juzenas et al., 2020)
miR-92a	Angiogenesis regulators	Enriched in RBC-derived EVs; implicated in cell–cell signaling and endothelial modulation	(Bonauer et al., 2009)
let-7i-5p	Parasite transcripts; endothelial inflammatory genes	Translocates into parasite cytoplasm to inhibit translation; EV-delivered let-7i-5p attenuates heme-induced endothelial inflammation	(LaMonte et al., 2012; Thomas et al., 2022)

### 2.3 miR-451a and oxidative stress during *Plasmodium* infection

MicroRNA-451a has emerged as a master regulator of red blood cell development and has essential roles in promoting erythroid differentiation, protecting against oxidative stress, and maintaining erythroid homeostasis. miR-451a primarily functions by repressing 14-3-3ζ and modulating autophagy pathways, and its expression is tightly controlled by the master erythroid transcription factor GATA-1. Evidence from cell lines, mouse models, and human clinical samples consistently demonstrates that microRNA-451a influences oxidative stress responses during *P. falciparum* infection. Joshi et al. examined the role of miR-451a in *P. falciparum*3D7-infected RBCs through transfection of a miR-451a mimic, which reduced parasitemia by ~50% within 48 h, alleviated oxidative stress, and restored red blood cell numbers, supporting miR-451a as a candidate therapeutic target (Yu et al., 2010; Joshi et al., 2024), further supporting a conserved role of miR-451a in redox regulation. Collectively, these findings suggest that miR-451a acts as a compensatory regulator of oxidative stress during malaria infection, maintaining red blood cell homeostasis and potentially restricting parasite growth by reinforcing FOXO3-dependent antioxidant pathways (Thomas et al., 2022). Along with let-7i-5p and miR-223, miR-451a can translocate into the parasite cytoplasm to inhibit translation (LaMonte et al., 2012). These miRNAs are notably enriched in sickle-cell erythrocytes, potentially contributing to their natural resistance to malaria. Circulating EVs that carry miR-451a and let-7i-5p also attenuate endothelial activation and inflammatory signaling *in vitro* (Thomas et al., 2022), suggesting systemic effects beyond red blood cells. Conversely, miR-16-5p, a housekeeping RBC miRNA, is consistently downregulated in the plasma of malaria patients (Chamnanchanunt et al., 2015), suggesting its potential as a biomarker. However, further studies are necessary to determine the molecular interactions of miR-451a with the parasite transcriptome and host–parasite dynamics. Additional research should explore the role of EVs in miRNA-mediated communication. Future work should optimize transfection, reduce off-target effects, and evaluate the safety and efficacy of miR-451a therapies *in vivo*, particularly using clinical *P. falciparum* isolates, to advance miRNA-based malaria treatments.

## 3 Mechanisms by which human miRNAs shape malaria progression

Throughout the *Plasmodium* life cycle, the most prominent miRNA signals are host microRNAs that are either (i) reprogrammed in infected tissues such as the liver and brain or (ii) exported via EVs from iRBCs to recipient cells such as endothelial cells. These alterations consistently target a limited set of host pathways, including interferon and cytokine signaling pathways; immune cell recruitment; endothelial activation; barrier integrity, including the blood–brain barrier; and EV-mediated intercellular communication, which can impact both disease progression and parasite transmission. **Table 2** summarizes the host pathways influenced by variations in miRNA profiles across different infection stages. Read across the table, two patterns stand out: liver-stage changes (miR-21, miR-155) act mainly on pre-erythrocytic immunity, whereas blood-stage and EV-borne miRNAs converge on a common set of endothelial and neuro-inflammatory pathways (barrier integrity, TGF-β/FoxO signaling, cytokine and interferon responses) that link the molecular changes to the organ-specific syndromes of severe malaria.

**Table 2:** Host pathways influenced by miRNA changes across infection stages.

Stage/compartment	miRNA(s)	Change	Targeted host pathway/effect	Model/evidence (ref.)
Liver stage	miR-21, miR-155	Up	Pre-erythrocytic immunity; CD8^+^ T-cell protection by attenuated parasites	Mouse *P. berghei*; antagomir KD + AAV8 overexpression (Hammerschmidt-Kamper, 2012; Hentzschel et al., 2014)
Erythrocytic/direct parasite targeting	miR-197-5p, miR-150-3p	Functional (mimic)	Hybridize to parasite apicortin mRNA → reduced growth, invasion, microneme (AMA1) secretion	(Chakrabarti et al., 2020)
Erythrocytic/host–parasite import	let-7a, miR-15a, miR-451, miR-140	Imported	Human AGO2–miRNA complexes enter parasite; miR-451/miR-140 down-regulate PfEMP1/var	*P. falciparum* (Wang et al., 2017; Dandewad et al., 2019)
Endothelium (vascular)	EV-borne host miRNAs (AGO2-bound)	Transferred	Silence CAV1, ATF2 → reduced TEER, increased permeability, junction/cytoskeleton disruption	iRBC-EVs → endothelial cells (Mantel et al., 2016)
Endothelium (tissue-specific)	Distinct brain vs. lung miRNA/mRNA sets	Altered	Cell-type-specific endothelial reprogramming after iRBC exposure	Brain vs. lung EC (Metwally et al., 2024)
Brain/cerebral malaria	let-7i, miR-27a, miR-150; miR-19a/b-3p, miR-142-3p, miR-223-3p	Up	TGF-β, FoxO, adherens junctions, endocytosis; miR-142-3p suppresses VE-cadherin (cdh5) → BBB dysfunction	ECM, PbA in CBA mice (El-Assaad et al., 2011; Cohen et al., 2018; Martin-Alonso et al., 2018)
Immune/EV cargo	miR-451a; miR-146a, miR-193b (MVs)	Altered	Chemokine/IFN targets (CXCL10, CCL5, IL7, OAS1); EV-miR-451a may dampen neutrophil ROS/cytokine output	Patient EVs; ECM MVs (Babatunde et al., 2017; Cohen et al., 2018; Ketprasit et al., 2020)
Plasma (*P. vivax*)	miR-223-3p, miR-145, miR-155; miR-7977, miR-28-3p, others	Up	Severity-correlated circulating signature; candidate prognostic biomarkers (AUC ~0.7)	Human cohorts (Kaur et al., 2018; Hadighi et al., 2022)

### 3.1 Direct parasite targeting

Host miRNAs can directly target parasite transcripts through various mechanisms. One such mechanism involves the transfer of host-to-parasite miRNAs, in which human Argonaute 2 complexes containing specific miRNAs, such as let-7a and miR-15a, are imported into *P. falciparum* (Dandewad et al., 2019). These complexes associate with parasite transcripts, facilitating host miRNA-mediated regulation of parasite gene expression. For example, miR-197-5p and miR-150-3p directly hybridize to the parasite apicortin mRNA, resulting in reduced parasite growth and invasion. The functional outcomes of this process include decreased expression of parasite proteins, such as apicortin; impaired parasite invasion; reduced microneme secretion (notably AMA1); and an overall decline in parasite fitness (Mantel et al., 2016; Chakrabarti et al., 2020).

#### 3.1.1 Parasite responses and possible counterstrategies

Host-to-parasite miRNA regulation is unlikely to be a one-way process, and a balanced view must consider how the parasite might tolerate or evade it. Because *Plasmodium* lacks a canonical RNA-interference machinery, it has no recognizable Dicer or Argonaute ortholog, imported human miRNA–AGO2 complexes act in a fundamentally foreign cytoplasmic environment, and the durability of their silencing is set by the host complexes rather than amplified by a parasite RISC. Several adaptive routes are therefore plausible and merit explicit study: (i) sequence variation or codon-usage bias in target transcripts that weakens seed-region complementarity, analogous to how *var* gene diversity underlies antigenic variation; (ii) stage-restricted expression of vulnerable transcripts, limiting the window during which an imported miRNA can act; (iii) modulation of the abundance or composition of erythrocyte-derived vesicles taken up by the infected cell; and (iv) parasite-driven remodeling of the host erythrocyte miRNA pool itself, as suggested by the observation that infection reshapes red-cell and EV miRNA profiles. We note that direct experimental evidence for these counterstrategies in *Plasmodium* is still lacking; distinguishing genuine parasite evasion from passive tolerance of an inert imported complex is an important open question and a prerequisite for any therapy that relies on host miRNAs to suppress parasite genes.

### 3.2 Host pathway modulation

#### 3.2.1 Endothelial and vascular effects

*Plasmodium falciparum* infection can impair endothelial function in part through miRNA-mediated regulation at the vascular interface. A key example of this mechanism involves extracellular EVs from *P. falciparum*-iRBCs. These EVs contain host miRNAs bound to AGO2, which results in the formation of RISC complexes. When internalized by endothelial cells, they silence specific genes such as CAV1 and ATF2, resulting in barrier disruption, as evidenced by decreased transendothelial electrical resistance, increased dextran permeability, and alterations in junctions and the cytoskeleton. Using miRNA “sponge” inhibitors can reduce these effects, indicating causality (Mantel et al., 2016). In addition to host miRNAs, small RNA sequencing of iRBC-EVs revealed a complex RNA payload (host and parasite RNAs) and demonstrated the transfer of iRBC-miRNAs to human endothelial cells, suggesting that EVs are a route for parasite–host communication that may influence endothelial defense and barrier properties (Babatunde et al., 2017). Consistent with direct endothelial reprogramming, an endothelial cell study reported cell type-specific changes in miRNA/mRNA profiles in brain versus lung endothelial cells after exposure to *P. falciparum*-iRBCs, supporting the idea that malaria triggers tissue-specific endothelial miRNA responses linked to early host–parasite interaction pathways (Metwally et al., 2024).

#### 3.2.2 Neuroinflammatory pathways

Brain-specific miRNAs (let-7i, miR-27a, and miR-150) are upregulated in cerebral malaria. Target pathways include TGF-β, FoxO, adherens junctions, and endocytosis and contribute to blood–brain barrier dysfunction and neuroinflammation (El-Assaad et al., 2011). Evidence from experimental cerebral malaria (ECM) models indicates that a distinct set of brain-expressed microRNAs becomes dysregulated during neurological disease, consistent with roles in neuroinflammation and blood–brain barrier (BBB) dysfunction. In CBA mice infected with *P. berghei* ANKA (ECM) versus non-cerebral malaria controls, microarray screening followed by qPCR validation revealed increased brain abundance of miR-19a-3p, miR-19b-3p, miR-142-3p, and miR-223-3p; pathway analyses linked these miRNAs to processes relevant to the ECM, such as endocytosis and adherens junction regulation; and a study highlighted a plausible BBB mechanism whereby elevated miR-142-3p can suppress vascular endothelial cadherin (VE-cadherin/cdh5) and weaken endothelial cell–cell adhesion and vascular integrity (Cohen et al., 2018; Martin-Alonso et al., 2018). Similarly, profiling of plasma microvesicles alongside brain tissue during ECM revealed dysregulated miRNA cargo (notably miR-146a and miR-193b in microvesicles) and mapped these signals to inflammatory cytokine production and cell–endothelium interaction pathways within the malaria KEGG framework-biological programs tightly coupled to cerebral vascular inflammation and neurological sequelae (Barker et al., 2017; Cohen et al., 2018).

#### 3.2.3 Immune system modulation

In *P. falciparum* infection, iRBCs and their EVs exhibit modified human miRNA profiles, and the predicted targets of infection-associated miRNAs include immune-relevant genes involved in chemokine/cytokine and interferon pathways (e.g., CXCL10, CCL5, IL7, OAS1), consistent with a role in immunomodulation (Wu et al., 2023). Mechanistically, iRBC-derived EVs were shown to contain functional AGO2–miRNA (RISC) complexes that are internalized by recipient cells and can silence target genes. In endothelial cells, this finding showed a direct link between EV–miRNA cargo and inflammation-associated vascular dysfunction in malaria (Mantel et al., 2016). EV/miRNA transfer may also influence immune evasion and pathology indirectly by regulating parasite virulence programs: RBC-derived microparticles carrying AGO2-bound miRNAs can be transferred into parasites, where host miRNAs (e.g., miR-451 and miR-140) were reported to downregulate the expression of *Pf*EMP1/*var*, a key determinant of sequestration and inflammatory disease severity (Chapman et al., 2017; Wang et al., 2017). In support of broader relevance, reviews of parasitic diseases emphasize that EV-associated RNAs can have immunomodulatory effects by triggering or suppressing host responses (Maurya and Sebastian, 2024), and a conference report specifically suggested that malaria-related EVs delivering miR-451a can dampen neutrophil antimicrobial functions (reduced ROS/cytokine output), potentially increasing susceptibility to bacterial coinfection (Babatunde et al., 2017; Ketprasit et al., 2020).

Repeated exposure to *P. falciparum* drives epigenetic reprogramming characterized by increased methylation of histone H3 at lysine 27 (H3K27me3), dimethylation of arginine residues, and decreased abundance of the histone variant H3.3 in monocytes and macrophages, all of which are modifications that correlate with suppressed proinflammatory cytokine responses (including TNF-α, IL-6, and IL-1β) and predict long-term disease tolerance. This trained tolerance phenotype, marked by elevated IL-10 production and regulatory chromatin landscapes enriched for H3K27me3 at inflammatory gene loci, enables asymptomatic parasitemia by dampening excessive inflammation while maintaining sufficient parasite control, thereby contributing to non-sterilizing immunity that allows chronic low-level infection without clinical symptoms. These studies do not examine miRNA mediators or establish causality for miRNA gain- or loss-of-function in those marks, raising questions about the role of miRNAs in immune modulation (Schrum et al., 2018; Crabtree et al., 2022; Dobbs et al., 2022).

## 4 Human miRNA profiles in other *Plasmodium* species

### 4.1 Plasmodium vivax

Limited data are available, indicating a need for further research into species-specific miRNA patterns. The most consistent *P. vivax*-related microRNA signaling pathway identified thus far is a circulating (plasma) miRNA profile in hosts that varies with infection and correlates with disease severity. In an Iranian cohort, RT–qPCR revealed higher levels of miR-223-3p, miR-145, and miR-155 in *P. vivax* patients than in controls, with fold changes of approximately 5.6, 16.9, and 1.7, respectively. These findings support the potential of these genes as biomarkers for disease prognosis (Hadighi et al., 2022). Extending this biomarker approach to severe cases, a microarray screening followed by qRT–PCR validation in complicated *P. vivax* malaria identified several miRNAs, hsa-miR-7977, hsa-miR-28-3p, hsa-miR-378-5p, hsa-miR-194-5p, and hsa-miR-3667-5p, as significantly upregulated. These factors showed moderate diagnostic performance (AUC ~ 0.7), with network and pathway analyses highlighting hubs such as UBA52 and pathways such as TGF-β signaling (Kaur et al., 2018). Additionally, some studies have used *in silico* methods to predict putative *P. vivax*-encoded pre-miRNA hairpins from the parasite genome (e.g., chromosomes 1–4). However, these candidates are purely computational predictions and require experimental validation to be confirmed as genuine parasite miRNAs (Sarker and Maulik, 2012).

### 4.2 *Plasmodium berghei* (rodent model)

In the *P. berghei* model, the miRNA signals specific to *P. berghei* described in previous research mainly reflect host miRNAs that change expression during infection rather than miRNAs produced by the parasite itself. In a *P. berghei* ANKA mouse model, the observed decrease in erythrocyte miRNA (miR-451) at high parasitemia was attributed to dilution by parasite RNA rather than infection-driven regulation (El-Assaad et al., 2011). Conversely, infection with *P. berghei* ANKA (PbA), which induces experimental cerebral malaria, was linked to a distinct brain miRNA signature: in CBA mice, qRT–PCR revealed that let-7i, miR-27a, and miR-150 were significantly upregulated in the brain during PbA infection but not during non-cerebral *P. berghei* K173 infection. This finding supports a role for host miRNA regulation in the inflammatory and neurovascular cascades that cause cerebral pathology (El-Assaad et al., 2011). Host miRNA modulation also appears to be important during the liver stage of *P. berghei* infection: transcript and miRNA profiles of infected mouse livers revealed broad changes, highlighting miR-21 and miR-155 as potential players in immune responses during immunization with attenuated parasites (Hammerschmidt-Kamper, 2012).

## 5 Drug development considerations (miRNA-based therapies, challenges, and chances)

miRNA-based therapies, encompassing replacement and inhibition strategies, are emerging as promising approaches. miRNA mimics are synthetic analogs engineered to compensate for lost miRNAs during infection and regulate critical immunological pathways. Conversely, miRNA inhibitors (antimirs) are antisense oligonucleotides that target overexpressed inflammatory miRNAs, thereby mitigating the inflammatory response. Both miRNA mimics and inhibitors face challenges related to delivery mechanisms, blood stability, and off-target effects. To overcome these barriers, innovative delivery systems, such as nanoparticles and liposomes, are being developed to efficiently deliver miRNA-based drugs to the site of infection while minimizing systemic toxicity. Furthermore, understanding the context-dependent functions of miRNAs is crucial for optimizing their therapeutic application. The development of miRNA mimics and inhibitors as therapeutic agents has shown promising results in preclinical and early clinical trials, and ongoing research has focused on refining delivery systems and improving specificity to ensure clinical success (Ishida and Selaru, 2013; Baumann and Winkler, 2014; Ganju et al., 2017; Ho et al., 2022; Chowdhury, 2024; Martino et al., 2025).

Direct antiparasitic approaches: miR-197-5p and miR-150-3p mimics exhibit direct antiparasitic effects by targeting the parasite apicortin and other essential proteins; thus, they could be used in combination therapy with conventional antimalarials (Chakrabarti et al., 2020).

Host-directed therapies: These include modulating host miRNA levels to enhance antimalarial immunity, targeting pathological miRNA signatures in severe malaria, and restoring beneficial miRNA functions (Ishida and Selaru, 2013).

Cross-kingdom miRNA/amiRNA approaches: Plant small RNAs can move across kingdoms and be deployed against pathogens via host expression, topical application, or engineered carriers, but direct evidence for SIDT-mediated uptake into *Plasmodium* is lacking. The amiRNA and nanoparticle approach shows efficacy in plants/fungi and could theoretically target the PfK13/PI3K pathways; however, its delivery into intraerythrocytic parasites and *in vivo* therapeutic efficacy have not been validated. Targeting the PfK13/PI3K pathway and proteostasis pathways is mechanistically rational given their role in artemisinin resistance, but the application of plant-derived miRNAs or amiRNAs to blood-stage *Plasmodium* currently lacks direct experimental support; focused proof-of-principle studies on uptake and intraparasitic activity are essential before therapeutic claims can be made (Chowdhury, 2024).

### 5.1 Delivery systems and stability challenges of miRNA-based therapies

However, successful miRNA therapeutics face various challenges, including instability in the bloodstream and limited cellular uptake. To overcome this challenge, delivery methods such as lipid nanoparticles (LNPs), polymeric nanoparticles, and exosomes have been used. LNPs protect miRNAs from degradation, enable controlled release, and can be targeted with ligands for precision, showing success in RNA therapies and cancer models. Polymeric nanoparticles such as PLGA respond to stimuli to enable targeted, controlled release, thereby improving efficacy. Exosomes are naturally involved in cell communication and efficiently and biocompatibly transport miRNAs; they have engineering potential but face production challenges. For stability and binding affinity, locked nucleic acids (LNAs) resist degradation and improve target binding. CRISPR–Cas9 allows precise genomic editing of miRNAs for durable regulation, and miRNA sponges can sequester harmful miRNAs, enhancing nanoparticle delivery stability and uptake (Ho et al., 2022; Seyhan, 2024; Li et al., 2025; Martino et al., 2025; Wang et al., 2025).

### 5.2 Off-target effects and toxicity

miRNA-based therapies using mimics and inhibitors show promise for cancer treatment but also pose risks such as off-target effects, immune responses, and toxicity. Off-target effects occur when miRNAs bind unintended genes, affecting multiple pathways. Chemical modifications for stability, such as 2’-O-methyl or LNA, can also elicit immune responses by activating Toll-like receptors, thereby inducing inflammation and tissue damage. Immune responses are another critical concern, as miRNA-based therapies can inadvertently activate both local and systemic immune reactions. Delivery systems, particularly lipid-based nanoparticles, can trigger the complement system, leading to hypersensitivity reactions and, in severe cases, anaphylaxis, a phenomenon known as complement activation-related pseudoallergy (CARPA). Innate immune activation can also occur, as some RNA molecules are recognized by Toll-like receptors (TLRs), leading to the release of proinflammatory cytokines. To mitigate these issues, researchers are investigating immune-compliant strategies, such as coating nanoparticles with polyethene glycol (PEG), to reduce immunogenicity; however, these modifications can reduce delivery efficiency and may introduce additional challenges (Bak and Mikkelsen, 2014; Catela Ivkovic et al., 2017; Ganju et al., 2017; Moles, 2017; Rupaimoole and Slack, 2017; Mollaei et al., 2019; Abdelaal and Kasinski, 2021; Zhang et al., 2021; Diener et al., 2022; Paunovska et al., 2022; Zhou et al., 2023; Brillante et al., 2024; Avendaño-Portugal et al., 2025; Bereczki et al., 2025; Rurik et al., 2025; Álvarez-Corrales et al., 2026; Tuteja et al., 2026).

Outside the malaria field, the development of miRNA-based therapies includes anti-miR-122 compounds that have reached phase II trials for hepatitis, but no miRNA drug has been approved yet. Issues with toxicity, off-target effects, and delivery challenges have led to trial pauses and ongoing optimization of chemical and targeting methods. miR-34 mimics, currently in phase I trials for cancer treatment, serve as examples of replacement strategies. Additionally, PEG-liposome delivery of miR-122 improved outcomes in rodent stroke models, indicating the potential for systemic treatment and therapeutic timing. Moreover, AAV9-mediated miRNA expression demonstrated sustained target suppression in a CMT1A model, supporting the feasibility of intrathecal viral delivery for treating genetic neuropathies (Catela Ivkovic et al., 2017; Mollaei et al., 2019).

## 6 Concluding remarks and future perspectives

**Figure 3:**
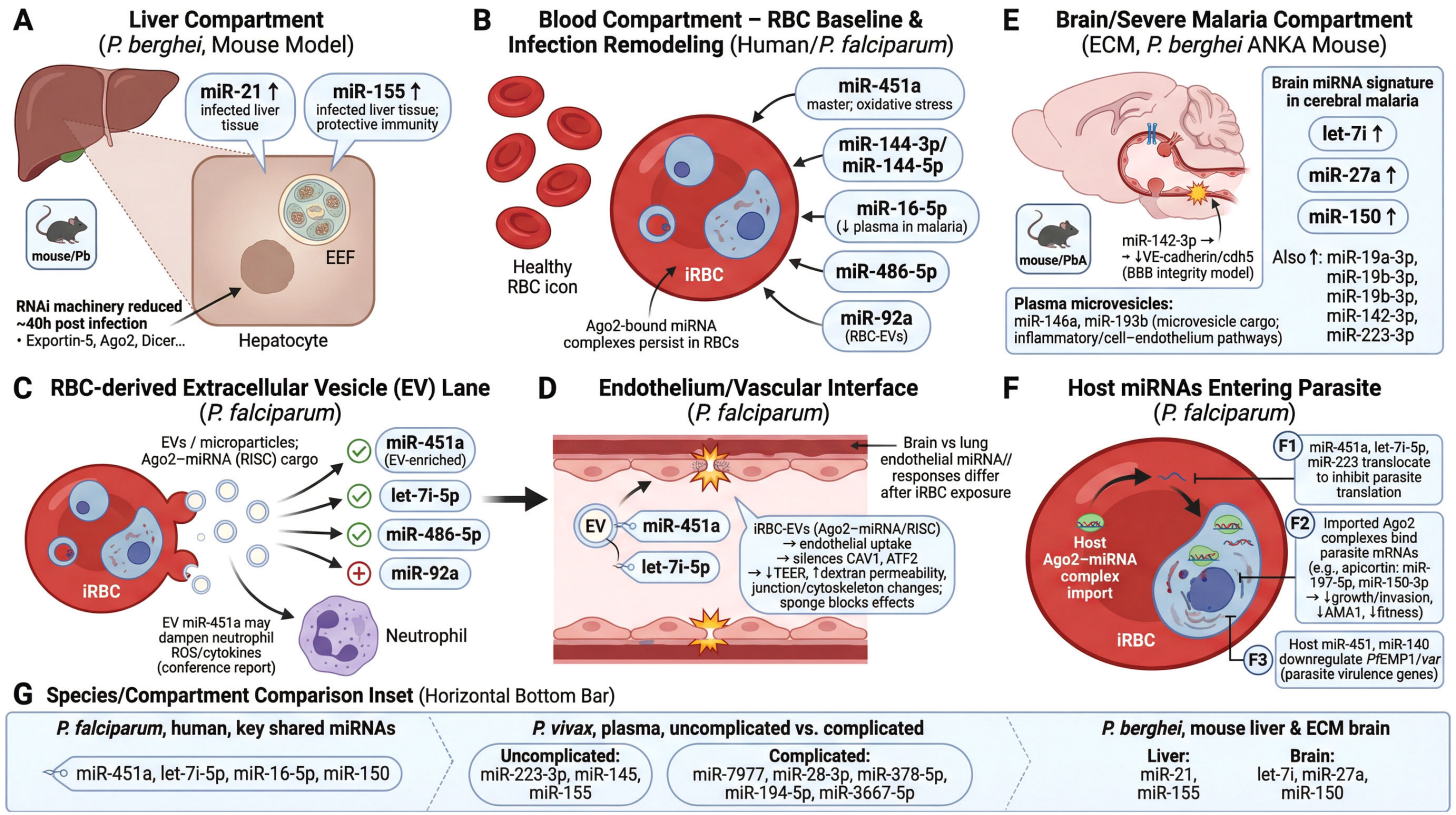
Integrated overview of host miRNAs implicated in malaria, by compartment. This schematic summarises the principal host miRNAs and their functional consequences across the major host compartments and the host–parasite interface; panels are labelled A–G. **(A)** Liver compartment (*P. berghei*, mouse model): miR-21 and miR-155 are up-regulated in infected liver tissue, with miR-155 associated with protective immunity, while host RNAi machinery (Exportin-5, Ago2, Dicer) is reduced ∼40 h post-infection within the hepatocyte, where the exoerythrocytic form (EEF) develops. **(B)** Blood compartment—red blood cell (RBC) baseline and infection remodelling (human/*P. falciparum*): dominant erythrocyte miRNAs include miR-451a (master regulator; oxidative stress), miR-144-3p/miR-144-5p, miR-16-5p (decreased in plasma during malaria), miR-486-5p and miR-92a, and Ago2-bound miRNA complexes persist in infected RBCs (iRBCs). **(C)** RBC-derived extracellular vesicle (EV) lane (*P. falciparum*): EVs/microparticles carry Ago2–miRNA (RISC) cargo enriched for miR-451a, let-7i-5p and miR-486-5p (miR-92a not enriched); EV-associated miR-451a may dampen neutrophil reactive oxygen species and cytokine responses. **(D)** Endothelium/vascular interface (*P. falciparum*): iRBC-EV delivery of miR-451a and let-7i-5p to endothelial cells silences CAV1 and ATF2, lowers trans-endothelial electrical resistance (TEER) and increases dextran permeability with junctional and cytoskeletal changes; brain versus lung endothelial responses differ after iRBC exposure. **(E)** Brain/severe-malaria compartment (experimental cerebral malaria, ECM; *P. berghei* ANKA mouse): the cerebral miRNA signature includes up-regulated let-7i, miR-27a and miR-150 (and miR-19a-3p, miR-19b-3p, miR-142-3p and miR-223-3p); miR-142-3p reduces VE-cadherin (cdh5) in a blood–brain-barrier integrity model, and plasma microvesicles carry miR-146a and miR-193b. **(F)** Host miRNAs entering the parasite (*P. falciparum*): host Ago2–miRNA complexes are imported into the iRBC, where (F1) miR-451a, let-7i-5p and miR-223 translocate to inhibit parasite translation; (F2) imported Ago2 complexes bind parasite mRNAs (e.g., apicortin; miR-197-5p and miR-150-3p), reducing growth and invasion (↓AMA1, ↓fitness); and (F3) host miR-451 and miR-140 down-regulate *Pf*EMP1/*var* virulence genes. **(G)** Species/compartment comparison inset (bottom bar): key shared miRNAs for *P. falciparum* in humans (miR-451a, let-7i-5p, miR-16-5p, miR-150); *P. vivax* plasma miRNAs distinguishing uncomplicated (miR-223-3p, miR-145, miR-155) from complicated disease (miR-7977, miR-28-3p, miR-378-5p, miR-194-5p, miR-3667-5p); and *P. berghei* mouse liver (miR-21, miR-155) versus ECM brain (let-7i, miR-27a, miR-150). Arrows indicate the direction of effect and ↑/↓ denote up-/down-regulation.

Host miRNAs play critical and multifaceted roles during *Plasmodium* infection (**Figure 3**), serving as both regulators of host–parasite interactions and mediators of pathological processes. The comprehensive mapping presented in this report revealed the following:

Extensive miRNA reprogramming occurs during *Plasmodium* infection, triggering widespread alterations in host miRNA expression. Specific host miRNAs, such as miR-197-5p and miR-150-3p, can directly target parasite transcripts, inhibiting parasite growth and invasion. Additionally, altered miRNAs modulate critical host pathways, including vascular function, immune responses, and neuroinflammation. The clinical potential of miRNA signatures is significant, as they show promise as biomarkers for disease severity and as therapeutic targets for both direct antiparasitic and host-directed interventions. However, miRNA responses are species- and stage-specific, varying by *Plasmodium* species, infection stage, and host organism, which necessitates tailored approaches for different clinical scenarios. miRNA mimics and inhibitors have therapeutic promise but face challenges such as off-target effects, toxicity, and immune responses. Improving delivery, reducing immune activation, and enhancing targeting are vital for safe, effective therapies. Studying changes in host miRNAs during *Plasmodium* infection offers insights into the molecular mechanisms of malaria and potential new treatments.

Mechanistic priorities focus on characterizing miRNA–target interactions, monitoring miRNA dynamics during infection, identifying species-specific responses, and understanding stage-specific regulation. For clinical use, efforts should standardize miRNA biomarker detection protocols, validate them across populations, conduct clinical trials, and develop regulatory pathways.

Finally, there are still some unresolved questions that need to be addressed:

1. Causality versus correlation: Many studies have shown that miRNAs are differentially expressed during infection, but it is unclear which changes drive pathology or protection and which are by-products of inflammation, hemolysis, or erythropoiesis.
2. Which cell types matter most, and when? Malaria involves multiple compartments: RBCs, endothelium, liver, and immune cells. A gap exists in mapping cell type-specific miRNA programs across stages and in linking them to outcomes such as fever, inflammation, cytoadhesion, vascular dysfunction, and anemia. Spatial transcriptomics and single-cell multiomics may advance the understanding of miRNAs in cerebral malaria by enabling detailed spatial, cell-type-specific, and integrative analyses. In cerebral malaria, transcriptional profiling has identified distinct brain niches with activated inflammatory signatures, microglia with T cells, and interferon-γ neurons, all of which are potential sites of miRNA regulation. Technologies such as Spectrum-FISH measure miRNAs across brain layers, while tools such as miTEA-HiRes infer miRNA activity from scRNA-seq and spatial data, allowing validation of regulatory activity against spatial miRNA distributions (Zhang et al., 2024; Chen et al., 2025).
3. EV biology: What are the “active ingredients” and transfer rules? EVs are involved in malaria pathogenesis and host–parasite communication, but major unknowns remain: which EV subtypes carry informative miRNA signals, how efficiently EV-associated miRNAs are delivered *in vivo*, and whether EV–miRNA signatures reprogram cells or reflect upstream processes.
4. Severe malaria and cerebral malaria: Are signatures conserved and valuable? Most EV–miRNA studies focus on uncomplicated malaria, but profiling should expand to severe cases such as anemia and cerebral malaria. It is important to determine whether the signatures are consistent across regions, ages, and comorbidities and to consider confounders, especially RBC-linked miRNAs. Candidate miRNAs such as miR-16, miR-155, miR-150, miR-451, and miR-223 are linked to malaria.
5. Do RBC miRNA shifts reflect parasite biology or host stress? Infection alters RBC and RBC-EV miRNAs, targeting immune pathways. An open question: Are these changes due to parasite remodeling or host stress, and which affects symptoms and transmission more?
6. Therapeutics: Can miRNA research go beyond biomarkers? Most of those studies focused on biomarkers, with limited progress in developing treatments and raising issues about target choice, delivery (including EV strategies), safety, and outcomes.

## Glossary

miRNA: microRNA
EVs: extracellular vesicles
MVs: microvesicles
RISC: RNA-induced silencing complex
AGO2: Argonaute 2
BBB: blood–brain barrier
CM: cerebral malaria
ECM: experimental cerebral malaria (mouse model)
RBC: red blood cell
iRBCs: infected red blood cells
*Pb*A: *Plasmodium berghei* ANKA
*Pf*EMP1: *P. falciparum*erythrocyte membrane protein 1
EPCR: endothelial protein C receptor
TF: tissue factor
TM: thrombomodulin
CAV1: caveolin-1
ATF2: activating transcription factor 2
VE-cadherin: vascular endothelial cadherin
cdh5: cadherin 5 gene (VE-cadherin)
UTR: untranslated region
qPCR: quantitative polymerase chain reaction
mRNA: messenger RNA
RNA: ribonucleic acid
PCR: polymerase chain reaction
ROS: reactive oxygen species
ATP: adenosine triphosphate
